# Association between FGFR1OP2/wit3.0 Polymorphisms and Residual Ridge Resorption of Mandible in Korean Population

**DOI:** 10.1371/journal.pone.0042734

**Published:** 2012-08-06

**Authors:** Jee Hwan Kim, Min Young Oh, Janghyun Paek, Jaehoon Lee

**Affiliations:** 1 Department of Prosthodontics, College of Dentistry, Yonsei University, Seoul, Korea; 2 Department of Prosthodontics, Kyung Hee University Dental Hospital, Seoul, Korea; National Institute of Environmental Health Sciences, United States of America

## Abstract

**Background:**

A previous study on the genetic association between single nucleotide polymorphisms in FGFR1OP2/wit3.0 and the long term atrophy of edentulous mandible hypothesized that the excessive jawbone atrophy after dental extraction may be associated with abnormal oral mucosa contraction induced by the FGFR1OP2/wit 3.0 gene. It was reported that the minor allele of rs840869 or rs859024 in FGFR1OP2/wit3.0 was associated with the excessive atrophy of edentulous mandible. The present study represents an attempt to replicate the results of this previous study and to examine the genetic association between polymorphisms in FGFR1OP2 and residual ridge resorption of mandible in a Korean population.

**Methodology/Principal Findings:**

134 subjects (70.46±9.02 years) with partially or completely edentulous mandible were recruited. The mandibular bone height was measured following the protocol of the American College of Prosthodontists (ACP). From 24 subjects, seven variants in FGFR1OP2 were discovered and four of them were novel. Selected SNPs that are not in high LD at r2 threshold of 0.8 were genotyped for the remaining population. There was no frequency of the minor allele of SNP rs859024 in Korean population. SNP rs840869 was not associated with residual ridge resorption (p = 0.479). The bone height of the subject with the ss518063493 minor allele (8.52 mm) was shorter than that of those subjects with major alleles (18.96±5.33 mm, p = 0.053).

**Conclusions/Significance:**

The patient with minor allele of ss518063493 may be associated with excessive atrophy of edentulous mandible whereas the patients with that of rs840869 are not associated in Korean population. The result from this study may assist in developing a novel genetic diagnostic test and be useful in identifying Koreans susceptible to developing excessive jawbone atrophy after dental extraction.

## Introduction

With the increase in human life expectancy, a great deal of effort has been put into improving quality of life. Reduced masticatory function from loss of teeth decreases quality of life and results in nutritional deficiency. When prosthodontic treatment is not given to the patient at an appropriate time, the resorption of the alveolar ridge may continue and result in a decrease in the masticatory efficacy of the prostheses and of patients' satisfaction. [1]–[5]

The residual alveolar ridge undergoes resorption most rapidly in the first 6 months, and its activity then continues at a slower rate. [6], [7] Many researchers have attempted to investigate the pathogenesis of resorption of the residual alveolar ridge, but they have not been able to show any significant results. Previous studies regarding the resorption of the alveolar ridge have been mainly focused on a gypsum model or radiological structural change in the residual alveolar ridge. [8] Atwood et al. suggested anatomic, prosthetic, metabolic, and functional factors as important etiologic agents of ridge resorption. [6] They tried to discover the cause of pathologic resorption, but no significant causes were found.

Studies quantitatively comparing alveolar ridge resorption which continues after dental extraction show a large difference among individuals. [9] Identifying high risk groups with severe resorption of residual alveolar ridge may provide helpful information in planning prosthodontic treatment. Our previous study showed the association between residual alveolar ridge resorption and single nucleotide polymorphisms (SNP) in fibroblast growth factor receptor 1 oncogene partner 2/wound inducible transcript 3.0 (FGFR1OP2/wit3.0). [10] Wit 3.0 is a cytoskeleton molecule that polymerizes and co-localizes with stress fibers, and it is upregulated by oral fibrolasts in dental extraction wounds. [11], [12] FGFR1OP2/wit3.0 overexpression increased the contraction of a fibroblast-populated floating collagen gel and full-thickness skin wounds in mice whereas heterozygous null mutation of FGFR1OP2/wit 3.0 decreased the migration rate of fibroblastic cells in vitro. [13] Hence, it has been assumed that FGFR1OP2/wit3.0 may be involved in the regulation of the accelerated oral wound contraction. [14] Based on this assumption, it was reported in our previous study that the edentulous oral mucosa may contribute to bone resorption which leads to residual ridge atrophy. During the early healing period after dental extraction, the gingival margins of the wound site contract toward the center of the extraction socket. The epithelium is rapidly integrated [15], [16] and regenerated into a small central part of the edentulous oral mucosa. [15] The edentulous oral mucosa is relatively thick with elongated rete pegs indicating acanthosis. [17], [18] On the contrary, the collagen density of the established residual ridge is increased whereas the connective tissue thickness is decreased in both denture wearers and non-denture wearers. These observations collectively indicate that the oral mucosa undergoes continuous remodeling and connective tissue contraction occurs. Consequently, the contraction may result in a thin oral mucosa and therefore be associated with the atrophic edentulous residual ridge.

The Wit 3.0 gene was initially isolated from mouse oral tissue. It is located on human chromosome 12 (26,982,583 to 27,010,129) and has 5 exons and 242 single nucleotide polymorphisms (SNPs) (GenBank, NCBI). There are a total of 7 cSNPs and rSNPs that affect genomic functional expression. Our previous study of the association of Wit 3.0 and alveolar ridge resorption in Caucasians in California, USA reported that there was statistical significance between alveolar ridge resorption and two SNPs in the Wit 3.0 gene (rs84086 and rs85902) [10]. This result was meaningful in showing that gene polymorphism is related with residual alveolar ridge resorption. However, this study had several limitations; in selecting the tag SNP, 6 assumed SNPs were randomly chosen from the Hap Map database that were sensitive for haplotype constructs (NCBI Build 36) instead of finding the variants of the gene from the DNA samples. Also, the selected SNPs did not represent ethnic or regional differences because they commonly appeared in Western European descendants, Han Chinese in Beijing, and Sub-Saharan Africans. SNPs are ethnicity specific, but this study did not consider ethnic differences in the association of SNPs with residual ridge loss since we did not choose SNPs directly from variant sites in patients' DNA samples. Another limitation was the small sample size, which increases the possibility of false positives.

The findings from our previous study may not be applicable in different ethnic groups and populations, and therefore it was important to conduct a similar association study with a larger sample size and better selection of SNPs in a different group. The purpose of this study was to examine the genetic association between single nucleotide polymorphisms (SNPs) of Wit3.0 and the excessive ridge resorption in edentulous mandible in a Korean population.

## Results

### Polymorphism discovery and genotyping the Wit 3.0 gene on chromosome 12q12.3

In a Korean population (n = 24), all exons, promoters, and exon-intron boundaries of the Wit 3.0 gene region on chromosome 12q12.3 were directly sequenced. Seven genetic variants were identified: four in promoter, one in intron, and two in coding regions as shown in Table 1. Of these polymorphisms, four were found to be novel by comparing our data with the dbSNP database Build 127 [http://www.ncbi.nlm.nih.gov/projects/SNP/]. Among these novel variants there were one insertion and one deletion; a dinucleotide CA repeat insertion was discovered in a promoter (ss518063498) and T deletion was discovered in an intron (ss518063913). The other novel variants were identified in exon 2 and exon 4 (ss518063493 and ss518063476).

**Table 1:** Genetic variants discovered in FGFR1OP2/wit3.0 gene by direct sequencing (n = 24).

Region	SNP number	SNP Name	Position	variation	Allele Frequency	HWE
Promoter	rs2306852	−17552A/G	chr12:27089540	A/G	A∶G = 0.979∶0.021	1
Promoter	rs2279351	−16542 A/C	chr12:27090550	A/C	A∶C = 0.938∶0.063	1
Promoter	ss518063498	−16161∼16150CA/−	chr12:27090931–27090942	CA/INS	[6]∶[7] = 0.958∶0.042*	1
Promoter	rs78054962	−15988C/T	chr12:27091104	C/T	T∶C = 0.958∶0.042	1
Exon2	ss518063493	E6E	chr12:27107109	G/A	G∶A = 0.979∶0.021	1
Intron2	ss518063913	IVS2+7∼11T/−	chr12:27107233–27107237	T/del	T∶- = 0.979∶0.021**	1
Exon4	ss518063476	M113T	chr12:27110618	C/T	T∶C = 0.979∶0.021	1

* [6] has six copies and [7] has seven copies of CA;

** T/del refer to [ttttt/tttt].

In our previous study, exon 5 in chromosomal DNA samples from various races was characterized by DNA direct sequencing in order to find two non-synonymous SNPs (rs1058701 and rs11613). It was reported that the over-expression of Wit 3.0 synthetic peptide carrying amino acid substitution by rs1058701 accelerates the rate of wound contraction in vitro and in vivo. [13] In this study, similarly, rs1058701 and rs11613 were observed in DNA samples from a Korean population by direct sequencing. As in our previous study, however, all subjects showed the homozygous wild type sequence and did not indicate the involvement of non-synonymous SNPs of exon 5 (data not shown).

For genotyping, rs840869, reported in our previous study, was added to our analysis to compare the result in a Korean population. The genotype frequency of each of the eight genetic variants studied is shown in Table 2 (7 SNPs from the current study and 1 SNP from the previous study). All of these variants were in Hardy-Weinberg equilibrium (p>0.05).

**Table 2:** Genotype frequencies of FGFR1OP2/wit 3.0 polymorphisms in 134 Koreans.

SNP number	A.A Change	Genotype				MAF	HWE
rs2306852	-	AA	GA	GG	N	0.004	1.000
		130	1	0	131		
rs2279351	-	AA	AC	CC	N	0.083	1.000
		112	20	1	133		
ss518063498	-	66	67	77	N	0.018	1.000
		128	5	0	133		
rs78054962	-	TT	CT	CC	N	0.038	1.000
		123	10	0	133		
ss518063493	E6E	GG	GA	AA	N	0.004	1.000
		130	1	0	131		
ss518063913	-	TT	T-	–	N	0.008	1.000
		132	1	0	133		
ss518063476	M113T	TT	CT	CC	N	0.007	1.000
		132	2	0	134		
rs840869*	-	CC	CG	GG	N	0.082	0.606
		112	22	0	134		

* SNP from previous study.

### Association of FGFR1OP2/wit 3.0 polymorphisms and residual ridge resorption

From the Student's t-test and ANOVA test results, the presence of minor allele (A) of a novel SNP in exon 2 (ss518063493) was not significant but strongly suggestive that it was associated with mandibular atrophy in dominant (p = 0.053) and codominant models (p = 0.053) (Table 3). ss518063493 is a synonymous SNP involving a G to A substitution with no Wit 3.0 amino acid sequence change. A subject with the A allele (8.52 mm) had shorter height of residual bone than those with the G allele (18.96±5.33 mm). All other SNPs were shown not to be associated with residual ridge resorption (RRR).

**Table 3:** Association of FGFR1OP2/wit 3.0 polymorphisms and mandibular height in 134 Koreans.

SNP Number	Model	p-value	Genotype	N	Mean(mm)	Std(mm)
rs2306852	Dominant	0.973	GG+GA	1	18.790	-
			AA	130	18.9780	5.492
	Recessive		GG	-	-	-
			AA+GA	131	18.976	5.470
	Codominant	0.973	AA	130	18.978	5.492
			GA	1	18.790	-
			GG	-	-	-
rs2279351	Dominant	0.451	CC+AC	21	18.080	4.875
			AA	112	19.065	5.574
	Recessive	0.271	CC	1	12.900	-
			AA+AC	132	18.955	5.459
	Codominant	0.472	AA	112	19.065	5.574
			AC	20	18.340	4.851
			CC	1	12.9000	-
ss518063498	Dominant	0.0812	77+67	5	23.132	4.261
			66	128	18.813	5.424
	Recessive		77	-	-	-
			66+67	133	18.975	5.434
	Codominant	0.0812	66	128	18.813	5.424
			67	5	23.132	4.261
			77	-	-	-
rs78054962	Dominant	0.104	CC+CT	10	16.182	4.652
			TT	123	19.080	5.434
	Recessive		CC	-	-	-
			TT+CT	133	18.862	5.418
	Codominant	0.104	TT	123	19.080	5.434
			CT	10	16.182	4.652
			CC	-	-	-
ss518063493	Dominant	**0.053**	AA+GA	1	8.520	-
			GG	130	18.957	5.332
	Recessive		AA	-	-	-
			GG+GA	131	18.877	5.389
	Codominant	**0.053**	GG	130	18.957	5.332
			GA	1	8.520	-
			AA	-	-	-
ss518063913	Dominant	0.342	--+T-	1	24.100	-
			TT	132	18.870	5.466
	Recessive		--	-	-	-
			TT+T-	133	18.909	5.464
	Codominant	0.342	TT	132	18.870	5.466
			T-	1	24.100	-
			--	-	-	-
ss518063476	Dominant	0.844	CC+CT	2	18.170	0.806
			TT	132	18.936	5.486
	Recessive		CC	-	-	-
			TT+CT	134	18.924	5.446
	Codominant	0.844	TT	132	18.936	5.486
			CT	2	18.170	0.806
			CC	-	-	-
rs840869	Dominant	0.479	GG+CG	22	18.169	5.814
			CC	112	19.073	5.396
	Recessive		GG	-	-	-
			CC+CG	134	18.924	5.446
	Codominant	0.479	CC	112	19.073	5.386
			CG	22	18.169	5.814
			GG	-		

## Discussion

The maintenance of residual ridge is essential for prosthodontic treatment. The height of the residual ridge is sustained when the alveolar bone is in balance with the surrounding tissues including the teeth, periodontal ligament, oral mucosa, and connective tissue. The volume and the shape of the alveolar process, a tooth-dependent tissue that develops in conjunction with the eruption of the teeth, undergo atrophy subsequent to removal of the teeth. [19] The bundle bone that anchors the tooth to the jaw loses its function and disappears. [20]–[22] The contraction of the connective tissue after the loss of teeth may create tension that disturbs the balance in bone remodeling and consequently leads to residual ridge resorption. The resorption processes in various tissues result in a narrower and shorter ridge. [23]

Tallgren's study demonstrated a high individual variability in mandibular bone loss after the extraction of teeth for edentulous patients. [9] A phenotypically complex trait like RRR is affected by various factors such as environmental factors, and little is known about the genetic tendency toward RRR. The SNP, a genetic marker with high accuracy, is commonly used for association studies of complex traits because one candidate gene carries many genetic variants and SNPs have a stable inheritance over generations.

When a tooth is surgically removed, a large void in the alveolar bone or the socket is created and undergoes a series of wound healing events. The bone socket initiates new bone apposition, and the external surface of the alveolar bone undergoes bone resorption, resulting in the generation of a saddle-shaped residual ridge. The unregulated expression of FGFR1OP2/wit 3.0 has been shown to significantly enhance cell migration and wound contraction. Because FGFR1OP2/wit 3.0 is involved in the wound healing of oral mucosa, it is likely that genetic variation in the gene may be involved with prolonged contraction leading to excessive atrophy of the mandible.

In our previous study, we reported that the dominant minor allele of SNPs rs840689 and rs859024 of FGFR1OP2/wit 3.0 may be associated with severely resorbed edentulous mandible. However, the possible involvement of ethnic diversity in the SNP genotypes in FGFR1OP2/wit 3.0 was not evaluated in the previous study. In the current study, we extensively examined FGFR1OP2/wit 3.0 gene region polymorphisms with a focus on genetic associations in a Korean population. Based on the Korean frequency data from the Korean Hapmap Project (http://www.khapmap.org/), there was no frequency of the minor allele of SNP rs859024 (A: G = 0:1), while rs840689 had 7% of MAF (C:G = 0.927: 0.073) in the Korean population. SNP rs840689 was selected for replicating previous results, but no correlation was shown in this study. It is assumed that the effect of the dominant minor allele of rs840689 may not be applicable for different populations, particularly Korean ones.

The present study did not demonstrate statistically significant SNPs that were associated with RRR in the Korean population. However, there was one subject with dominant minor allele of ss518063493 that fell into the ACP type IV group. The ACP classification Type IV refers to severely compromised edentulous ridges, whereas Type I, II, and III refer to minimally, moderately and substantially compromised edentulous ridges. Although this is not statistically supportive, it can be assumed that subjects, carrying the dominant minor allele of ss518063493, have a potential risk of developing atrophied edentulous mandible.

A larger number of subjects participated in the current study compared to previous studies (n = 20), but only a small number of the subjects had severely atrophied mandible and generally patients with ACP type IV are rare to find the population. Therefore, a several independent cohort studies in a longer period are required in order to verify the results of the association between the genetic variants and RRR. However, this study has demonstrated a possible cause of atrophic jawbone resorption in a Korean population. Also, it is meaningful that four novel variants were discovered specifically in Koreans and one novel variant was negatively associated with RRR.

The outcome of the current study introduces a future genetic diagnostic method that may provide clinicians with an objective measure for identifying patients predisposed to severe atrophy of jawbone structure and to determine selection of the most beneficial treatment for the maintenance of maxillary and mandibular alveolar bone structure.

## Materials and Methods

### Ethics Statement

All research involving human subjects or human data was approved by the Institutional Review Board of Yonsei University College of Dentistry and Kyung Hee University Dental Hospital (Yonsei IRB No. 2-2010-0022, KHUSD IRB 2011-01). All clinical investigation was performed in accordance with the Declaration of Helsinki. Written informed consent was obtained from all participants before taking part in this study.

### Study Population

The study population consists of 134 unrelated Korean individuals, 50 men and 84 women, who were recruited between January 2011 to June 2012 from Yonsei University Dental Hospital and Kyung Hee University Dental Hospital. The mean age of participants was (70.46±9.02 years). The men were aged between 50 to 88 (60.92±9.14 years), and the women were aged between 51 and 90 (72.21±8.91 years). Each subject was completely or partially edentulous for at least two years. The partially edentulous subjects were missing both maxillary and mandibular premolars and molars either unilaterally or bilaterally. The patients with the following criteria were included in the study: 1) no known systemic conditions that could affect bone conditions such as osteoporosis, other metabolic bone disease, or pituitary disease, 2) no history of bone transplantation, and 3) presenting a panoramic dental radiograph less than 2 years old. An oral examination was performed to confirm their edentulous condition.

### Measurement of mandibular residual ridge height

**Figure 1:**
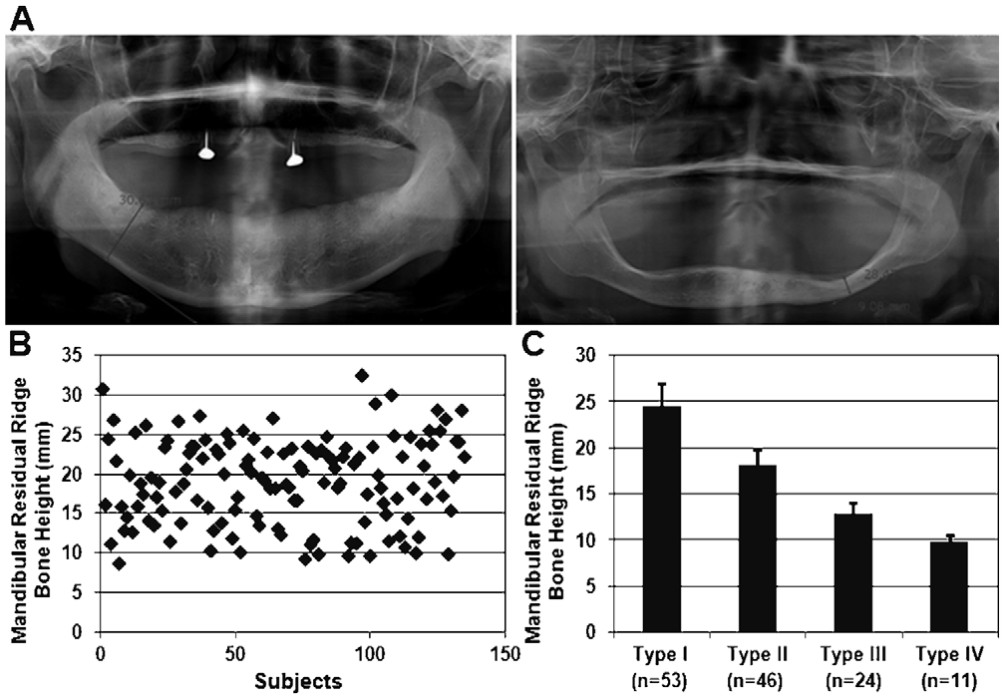
Characterization of mandibular ridge. A. Panoramic dental radiographs were used to determine the lowest residual ridge height (arrow) of edentulous mandible. Figure on the right indicates severe atrophy of edentulous mandible. B. Scatter plot of mandibular residual ridge height (n = 134). C. All subjects fell into one of the following ACP classifications: Type I (n = 53); Type II (n = 46); Type III (n = 24); Type IV (n = 11).

The panoramic dental radiograph of each subject was assigned an anonymous identification number and stored in a secured computer with password protection. The lowest height of the edentulous mandible was measured according to the Prothodontic Diagnosis Index classification from the American College of Prosthodontists (ACP) (Figure 1A). The mean mandibular bone height was 18.92±5.45 mm (n = 134) and it varied from 8.52 mm to 32.34 mm (Figure 1B).The ACP classification is as follows: Type I: residual ridge bone height of 21 mm or greater; Type II: residual ridge bone height of 16 to 20 mm; Type III: residual ridge bone height of 11 to 15 mm; and Type IV: residual ridge bone height of 10 mm or less. [24] Of our subjects, 53 were classified as Type I (mean residual ridge bone height 24.38±2.46 mm); 46 as Type II (18.06±1.69 mm); 24 as Type III (12.72±1.21 mm); and 11 as Type IV (9.75±0.65 mm) (Figure 1C).

### Genomic DNA extraction

Each edentulous subject was asked to collect 2 ml of saliva in the tube of an Oragene DNA Self-Collection Kit that contained 2 ml of DNA-preserving solution (DNA Genotek, Ottawa, Ontario, Canada, Cat. #OG-500). After the saliva was collected, the lid was closed and the liquid in the lid was released into the tube to mix with the saliva. The sample with a total volume of 4 ml was stored at 25°C for no longer than 2.5 weeks. Genomic DNA was collected using the Puregene DNA purification kit from Qiagene (Qiagene, Valencia, CA, USA) according to the manufacturer's protocol. DNA extractions and further analysis were performed by DNA Link Inc., Seoul, South Korea. The concentration of DNA was measured by the Nanodrop Spectrophotometer-1000 (Thermo Scientific, Delaware, USA).

### DNA Direct Sequencing

From the first 24 patients' DNA samples, the FGFR1OP2 gene was divided into 12 fragments and each fragment was amplified by using the PCR method. The final volume used for PCR was 10 µl, consisting of 10 ng of DNA, 0.5 uM of each primer pair, 0.25 mM dNTPs, 3 mM MgCl_2_, 1 µl 1×reaction buffer, and 0.25 U Taq DNA polymerase (Intron Biotechnology, Seongnam-Si, Gyeonggi-do, Korea). The PCR conditions used were as follows: initial denaturation at 94°C for 5 min, followed by 35 cycles of denaturation at 94°C for 30 s, annealing at 60–65°C for 30 s, initial extension at 72°C for 30–60 s, and final extension at 72°C for 10 min. The PCR products were purified using a MultiScreen384-PCR Filter Plate (Millipore, Billerica, MA, USA). The purified products were then sequenced using a BigDye Terminator Cycle Sequencing Kit and an ABI 3730xl automated sequencer (Applied Biosystems, Foster City, CA, USA). The sequencing primers were the same as those used for the PCR amplification. Mutation analyses were performed using the DNA analysis programs Phred, Phrap, Consed, and Polyphred 5.04 (http://droog.mbt.washington.edu/PolyPhred.html). For four novel variants discovered from DNA sequencing, we have deposited the raw data at GenBank under accession number ss518063913, ss518063476, ss518063493 and ss518063498. Direct sequencing was repeated several times to confirm that identified variants are valid.

### SNP selection

The identified variant sites (MAF>1%) from the first 24 patients and rs840869 reported in our previous study were used for constructing a haplotype and linkage disequilibrium (LD) block with Haploview [25] 8 SNPs that were not in high LD at r^2^ threshold of 0.80 in the FGFR1OP2 gene (rs2306852, rs2279351, ss518063498, rs78054962, ss518063493, ss518063913, ss518063476, rs840869) were chosen for genotyping.

### SNP genotyping-SNaPshot assay/Direct sequencing

2 SNPs (rs78054962, ss518063498) in the FGFR1OP2 gene were genotyped using direct sequencing and the remaining of 6 SNPs (rs2306852, rs2279351, ss518063493, ss518063913, ss518063476, rs840869) were genotyped using single base primer extension assay (SNaPshot) in all subjects. The genotyping was screened using a ABI PRISM SNaPShot Multiplex kit (ABI, Foster City, CA, USA) according to the manufacturer's instructions. The genomic DNA flanking each tSNP was amplified with PCR reaction with Forward and Reverse primer pairs and standard PCR reagents in 10 a microliter reaction volume, containing 10 ng of genomic DNA, 0.5 pM of each oligonucleotide primer, 1 microliter of 10× PCR buffer, 250 µM dNTP (2.5 mM each) and 0.25 unit DiaStar Taq DNA Polymerase (5unit/µl; SolGent Co., Ltd. Daejeon, South Korea). The PCR reactions were carried out as follows: 10 min at 95°C for 1 cycle, and 35 cycles at 95°C for 30 s, 60°C for 1 min, 72°C for 1 min followed by 1 cycle of 72°C for 10 min. After amplification, the PCR products were treated with 1 unit each of shrimp alkaline phosphatase (SAP) (USB Corporation, Cleveland, OH, USA) and exonuclease I (USB Corporation, Cleveland, OH, USA) at 37°C for 75 min and 72°C for 15 min to purify the amplified products. One microliter of the purified amplification products was added to a SNaPshot Multiplex Ready reaction mixture containing 0.15 pM of genotyping primer for the primer extension reaction. The primer extension reaction was carried out for 25 cycles of 96°C for 10 s, 50°C for 5 s, and 60°C for 30 s. The reaction products were treated with 1 unit of SAP at 37°C for 1 h and 72°C for 15 min to remove excess fluorescent dye terminators. One microliter of the final reaction samples containing the extension products were added to 9 microliters of Hi-Di formamide (ABI, Foster City, CA). The mixture was incubated at 95°C for 5 min, followed by 5 min on ice and then analyzed by electrophoresis in an ABI Prism 3730xl DNA analyzer. Analysis was carried out using Genemapper software (version 4.0; Applied Biosystems).

### Statistical analysis

All data analysis was completed using the statistics software SAS 9.1.3 (SAS Institute Inc. in Cary, NC, USA). Hardy-Weinberg equilibrium (HWE) tests were analyzed permutation test. The association of SNPs with mandibular jawbone atrophy was evaluated using t-tests for the dominant and recessive groups and ANOVA analysis for the codominant group. To estimate the extent of the pairwise linkage disequilibrium, the standard definition of D′ and r^2^ were calculated using the HaploView program version 4.2 (www.broadinstitute.org/haploview/haploview). D′ and r^2^ were calculated for polymorphisms with MAF>1%. A P-value less than 0.05 was considered statistically significant.
